# A retrospective analysis of the influencing factors of nucleic acid CT value fluctuation in COVID-19 patients infected with Omicron variant virus in Changchun city

**DOI:** 10.3389/fpubh.2024.1377135

**Published:** 2024-06-14

**Authors:** Zhenghua Cao, Feng Sun, Huan Ding, Zhiyu Tian, Yingzi Cui, Wei Yang, Shaodan Hu, Li Shi

**Affiliations:** ^1^Changchun University of Traditional Chinese Medicine, Changchun, Jilin, China; ^2^Affiliated Hospital of Changchun University of Traditional Chinese Medicine, Changchun, Jilin, China

**Keywords:** COVID-19, Omicron variant, fluctuation of nucleic acid CT values, retrospective analysis, risk factors

## Abstract

**Objective:**

This study aimed to determine the risk factors associated with fluctuations in nucleic acid CT values in patients infected with the Omicron variant during an outbreak at a hospital in Changchun city.

**Methods:**

A retrospective analysis was conducted on general information, medical history, vaccination history, and laboratory test data of COVID-19 patients infected with the Omicron variant and admitted to the hospital in Changchun from March 2022 to April 2022. The study aimed to explore the factors influencing nucleic acid CT value fluctuations in COVID-19 patients infected with the Omicron variant in Changchun city.

**Results:**

Fluctuations in nucleic acid CT values were significantly correlated with occupation composition (*p* = 0.030), hospital stay duration (*p* = 0.000), heart rate (*p* = 0.026), creatinine (*p* = 0.011), platelet count (*p* = 0.000), glutamic-pyruvic transaminase (*p* = 0.045), and glutamic oxaloacetic transaminase (*p* = 0.017). Binary logistic regression analysis revealed significant correlations between hospital stay duration (*p* = 0.000), platelet count (*p* = 0.019), heart rate (*p* = 0.036), and nucleic acid CT value fluctuations (*p* < 0.05), indicating that they were independent risk factors. Red blood cell count was identified as a factor influencing nucleic acid CT value fluctuations in Group A patients. Occupation composition, direct bilirubin, and platelet count were identified as factors influencing nucleic acid CT value fluctuations in Group B patients. Further binary logistic regression analysis indicated that occupational composition and direct bilirubin are significant independent factors for nucleic acid CT value fluctuations in Group B patients, positively correlated with occupational risk and negatively correlated with direct bilirubin.

**Conclusion:**

Therefore, enhancing patients’ immunity, increasing physical exercise to improve myocardial oxygen consumption, reducing the length of hospital stays, and closely monitoring liver function at the onset of hospitalization to prevent liver function abnormalities are effective measures to control fluctuations in nucleic acid CT values.

## 1 Introduction

In December 2019, the emergence of the novel coronavirus pneumonia (COVID-19, 2019-nCoV, or SARS-CoV-2) continued to perplex the entirety of human society. It has impacted not only the healthcare systems but also the equilibrium of the global socio-economic landscape (1, 2). The Omicron variant, known for its high transmissibility and immune evasion capabilities (3), can rapidly become the dominant strain in a region. However, it primarily infects the upper respiratory tract rather than the lower respiratory tractt (4), resulting in decreased pathogenicity and slower spread from the main bronchi to the distal bronchioles, leading to significant improvements in pulmonary histopathology and lung disease (5–7). The majority of patients may experience mild symptoms or remain asymptomatic (8, 9). Studies have indicated that Omicron possesses enhanced contagiousness and the ability to evade vaccine protection (10, 11). Real-time reverse transcription polymerase chain reaction (RT-PCR) can be employed for screening and diagnosing Omicron infections, closely associated with viral culture (12, 13). The virus may still be detected in individuals even 0–2 days after symptom resolution (14). Thus, monitoring the fluctuation of the patient’s nucleic acid cycle threshold (CT) values is crucial.

Over the past 3 years, there have been recurrent small-scale outbreaks of the pandemic, which have significantly affected the physical and mental health of the population. Research has revealed that a majority of individuals have developed a fear of the Omicron variant (15), with a temporary increase in emotional and anxiety disorders (16), leading to long-lasting psychological sequelae, albeit unevenly distributed (17). However, under the anti-pandemic measures implemented in China, it has been observed that the Chinese population’s fear of the Omicron variant has gradually diminished, while positive emotions have increased (18). As the number of infected individuals continues to rise, our clinical observations have indicated that fluctuations in the nucleic acid CT values of hospitalized COVID-19 patients can have an impact on their mood, potentially prolonging their recovery time and impacting their health. A plethora of systematic reviews and observational studies have established that nucleic acid CT values are not only correlated with the progression to severe illness, disease severity, and clinical biomarkers (19–21), but also bear significant associations with demographic factors and clinical histories (22–24). It has been observed that males exhibit higher viral loads compared to females (25), which can predict their infectiousness (26) and potentially reduce the likelihood of transmission (27). Consequently, CT values can serve as prognostic markers at an individual level (28, 29), identifying patients with higher morbidity and risk (30). Therefore, it is crucial to consider nucleic acid CT values in the management of COVID-19 infections (31), and to emphasize the importance of early testing (32). Further research indicates that fluctuations in CT values are linked with respiratory symptoms, comorbidities, and abnormalities in chest imaging (33), and may exacerbate the incidence of depression and insomnia (34). Prompt attention to the dynamic changes in CT values (35) will aid in monitoring the infection status of patients (36).

Therefore, this study aims to retrospectively analyze general information, medical history, vaccination records, laboratory tests, and other data of COVID-19 patients infected with the Omicron variant in Changchun, Jilin Province, China, in March 2022. Through statistical analysis of the collected data, we aim to explore the factors associated with fluctuations in patients’ nucleic acid CT values.

## 2 Materials and methods

### 2.1 General data of patients

The diagnostic criteria and fluctuation of nucleic acid CT values are referenced from the Diagnosis and Treatment Protocol for Novel Coronavirus Pneumonia (Trial Version 9) issued by the National Health Commission and the National Administration of Traditional Chinese Medicine (37). All COVID-19 patients undergo nucleic acid testing conducted by professionals at the same time and location, using specialized nucleic acid sampling techniques. The tests are uniformly processed using machines and reagent kits from the same batch and manufacturer. The Fluorescent Quantitative PCR method is employed, and results are interpreted strictly according to the kit’s specifications. The process includes initial screening, retesting, and CDC review to minimize potential errors. All test results adhere to the guidelines set forth in the “Diagnosis and Treatment Protocol for Novel Coronavirus Pneumonia (Trial Version 9)” issued by the National Health Commission and the National Administration of Traditional Chinese Medicine. The term “nucleic acid CT value fluctuation” refers to the scenario where a patient’s initial post-admission nucleic acid test is negative, marked as Day 1 with a CT value greater than 35, and the Day 2 test also shows a CT value greater than 35, but on Day 3, the CT value drops below 35, leading to a delayed discharge termed as nucleic acid CT value fluctuation.

### 2.2 Laboratory index and TCM diagnosis

Collection of general information, medical history, vaccination history, and laboratory examination data of COVID-19 patients who were hospitalized and infected with the Omicron variant at the Affiliated Hospital of Changchun University of Traditional Chinese Medicine from March 2022 to April 2022. Based on the results of the patients’ nucleic acid tests, they were divided into two groups: the nucleic acid CT value fluctuation group and the nucleic acid CT value non-fluctuation group. Patients were further divided based on the length of hospital stay: those hospitalized for more than 10 days were placed in Group A, while those hospitalized for 10 days or less were placed in Group B.

### 2.3 Statistical analysis

A database was constructed using WPS 2023 spreadsheet, and SPSS 20.0 was used for statistical analysis. Descriptive statistics such as frequency and percentage were used for categorical data, and the chi-square test was used for between-group comparisons. For normally distributed continuous data, mean and standard deviation were used for description, while non-normally distributed continuous data were described using median [P25, P75]. Logistic regression analysis was utilized to identify risk factors of nucleic acid CT value fluctuation. A significance level of *p* < 0.05 was considered statistically significant.

### 2.4 Ethical review

This study has been approved by the Ethics Committee of the Affiliated Hospital of Changchun University of Traditional Chinese Medicine (Approval No: CCZYFYLL2022 Approval Letter-020; CCZYFYLL2022 Approval Letter-021). All patients provided written or verbal informed consent prior to participation.

## 3 Results

### 3.1 General information of patients

This research collected data from 927 COVID-19 patients, categorizing them based on their nucleic acid test results into two groups: a stable CT value group with 660 cases and a fluctuating CT value group with 267 cases. Among the group with stable nucleic acid CT values, there were 388 males and 272 females, with a mean age of 40.27 ± 16.102 years. In the group with fluctuating nucleic acid CT values, there were 162 males and 105 females, with a median age of 41 [31, 53] years. There were no statistically significant differences between the two groups in terms of age, gender composition, vaccination history, medical history, and pre-admission treatment (*p* > 0.05). However, there was a statistically significant difference in the occupational composition between the two groups (*χ*^2^ = 7.043, *p* = 0.03) (Table 1).

**Table 1:** Comparison of basic information of patients [Example (%)].

Clinical data	Total (927)	Category		*χ*^2^/z	*p*
		Stable nucleic acid CT values (660)	Fluctuating nucleic acid CT values (267)		
Age		40.27 ± 16.102	41 [31,53]	−1.031	0.302
Sex				0.28	0.596
Male	550 (59.33)	388 (58.79)	162 (60.67)		
Female	377 (40.67)	272 (41.21)	105 (39.33)		
Occupation				7.043	0.03*
Low-risk occupation	88 (9.49)	52 (7.88)	36 (13.48)		
Medium risk occupation	454 (48.98)	327 (49.54)	127 (47.57)		
High-risk occupation	385 (41.53)	281 (42.58)	104 (38.95)		
Vaccine				0.000	0.988
Unvaccinated	59 (6.39)	42 (6.39)	17 (6.37)		
Vaccinated	865 (93.61)	615 (93.61)	250 (93.63)		
Previous history				3.261	0.071
Yes	130 (14.1)	84 (12.79)	46 (17.36)		
No	792 (85.9)	573 (87.21)	219 (82.64)		
Treatment before admission				0.178	0.673
No	827 (89.21)	587 (88.94)	240 (89.89)		
Yes	100 (10.79)	73 (11.06)	27 (10.11)		

*p < 0.05, the difference is statistically significant.

### 3.2 Laboratory indicators and traditional Chinese medicine (TCM) syndrome differentiation

There were statistically significant differences between the group with stable nucleic acid CT values and the group with fluctuating nucleic acid CT values in terms of hospital stay duration (*z* = −12.672, *p* = 0.000), heart rate (*z* = −2.224, *p* = 0.026), creatinine (*z* = −2.545, *p* = 0.011), platelet count (*z* = −3.502, *p* = 0.000), glutamic-pyruvic transaminase (*z* = −2.006, *p* = 0.045), and glutamic oxaloacetic transaminase (*z* = −2.388, *p* = 0.017). However, there were no statistically significant differences between the two groups in terms of TCM symptoms at admission, fever after admission, comprehensive evaluation of medical records, TCM diagnosis at discharge, and various indicators (temperature, breath, systolic blood pressure, diastolic blood pressure, oxygen saturation, total bilirubin, direct bilirubin, red blood cell count, white blood cell count, bacterial count, hemoglobin, albumin, blood urea nitrogen, NLR) (*p* > 0.05) (Tables 2, 3).

**Table 2:** Comparison of clinical data of patients after admission [P25, P75].

Clinical data	Total (927)	Category		*χ*^2^/z	*p*
		Stable nucleic acid CT values (660)	Fluctuating nucleic acid CT values (267)		
Hospital stay duration		10 [8,11]	12 [10,14]	−12.672	0.000*
TCM symptom				0.584	0.445
No	133 (14.35)	91 (13.79)	42 (15.73)		
Yes	794 (85.65)	569 (86.21)	225 (84.27)		
Fever after admission				2.810	0.094
Yes	192 (58.54)	129 (55.60)	63 (65.63)		
No	136 (41.46)	103 (44.40)	33 (34.38)		
Comprehensive evaluation of admission records				2.931	0.231
Light	790 (98.26)	570 (98.62)	220 (97.35)		
Ordinary	14 (1.74)	8 (1.38)	6 (2.65)		
Discharged TCM diagnosis				0.819	0.664
Lung meridian stagnation fire syndrome	520 (56.10)	365 (55.30)	155 (58.05)		
Evidence of cold and dampness invading outside	375 (40.45)	273 (41.36)	102 (38.20)		
Others	32 (3.45)	22 (3.33)	10 (3.75)		

*p < 0.05, the difference is statistically significant.

**Table 3:** Comparison of all indicators in patients [P25, P75].

Various indicators	Category		*z*	*p*
	Stable nucleic acid CT values (660)	Fluctuating nucleic acid CT values (267)		
Temperature	36.5 [36.2, 36.7]	36.5 [36.2, 36.7]	−0.221	0.825
Breath	18 [18, 20]	18 [18, 20]	−0.596	0.551
Heart rate	90 [80, 100]	88 [80, 100]	−2.224	0.026*
Systolic blood pressure	120 [120, 130]	120 [120, 129]	−0.283	0.777
Diastolic blood pressure	80 [74, 83]	80 [70, 82]	−0.673	0.501
Blood oxygen saturation	98 [96, 99]	98 [97, 99]	−0.003	0.998
Total bilirubin	10.3 [6.725, 13.975]	10.5 [7.5, 13.9]	−0.880	0.379
Direct bilirubin	2.9 [1.225, 4.0]	3.1 [1.8, 4.2]	−1.523	0.128
Creatinine	60.5 [41, 76]	66 [50, 78]	−2.545	0.011*
Red blood cell count	4.85 [1.8, 13.8]	4.8 [1.5, 12.4]	−0.630	0.529
White blood cell count	8.2 [3.6, 21.975]	7.0 [3.8, 16.8]	−1.122	0.262
Bacterial count	21.9 [4.6, 151.475]	13.4 [4.4, 141.2]	−0.928	0.353
Hemoglobin	144 [131, 158]	144 [130, 157]	−0.734	0.463
Platelet count	205 [169, 250]	189 [159, 235]	−3.502	0.000*
Albumin	42.05 [38.125, 44.175]	42.2 [39.3, 44.6]	−1.129	0.259
Glutamic-pyruvic transaminase	18 [9, 32]	21 (12, 34)	−2.006	0.045*
Glutamic oxaloacetic transaminase	20 [14, 26]	21 [16, 28]	−2.388	0.017*
Urea nitrogen	3.9 [2.7, 4.9]	4.0 [3.1, 4.9]	−1.143	0.253
NLR (Neutral Particle Count/Lymphocyte Count)	1.36 [0.94, 1.9775]	1.33 [0.85, 1.96]	−0.936	0.349

*p < 0.05, the difference is statistically significant.

### 3.3 Logistic regression analysis of factors influencing nucleic acid CT value fluctuations

Factors that showed statistically significant differences in the univariate analysis, including hospital stay duration, heart rate, creatinine, platelet count, glutamic-pyruvic transaminase and glutamic oxaloacetic transaminase, were included in a binary logistic regression model. Nucleic acid CT value fluctuation was the dependent variable, and the six aforementioned variables, along with age as a forced variable, were entered into the model using a forward: LR method. The results showed that hospital stay duration (*p* = 0.000, OR = 1.399), heart rate (*p* = 0.036, OR = 0.979), and platelet count (*p* = 0.019, OR = 0.996) were significantly associated with nucleic acid CT value fluctuations. None of the other indicators were independent risk factors for nucleic acid CT value fluctuations (*p* > 0.05) (Table 4).

**Table 4:** Logistic regression analysis of the fluctuation factors of nucleic acid CT value of patients.

Risk factors	OR (95%CI)	*p*
Hospital stay duration	1.399 (1.259–1.546)	0.000*
Age	0.213 (0.966–1.007)	0.164
Heart rate	0.979 (0.960–0.999)	0.036*
Platelet count	0.996 (0.993–0.999)	0.019*

*p < 0.05, the difference is statistically significant.

### 3.4 Comparison of baseline data between two groups

Since nucleic acid CT value fluctuations were positively correlated with hospital stay duration, the patients were further divided into Group A and Group B based on a median hospital stay duration of 10 days. In group A, there were 233 cases in the non-fluctuation group of nucleic acid CT value, including 139 males and 94 females, with a median age of 39 (30, 38) years, and 185 cases in the fluctuation group of nucleic acid CT value, including 115 males and 70 females, with a median age of 43 (31, 38) years. In group B, there were 427 cases in the non-fluctuation group of nucleic acid CT value, including 249 males and 178 females, with a median age of 39 (28, 38) years, and 82 cases in the fluctuation group of nucleic acid CT value, including 47 males and 35 females, with a median age of 34 (28, 39) years. There were no statistically significant differences between the two groups in terms of age, gender composition, vaccination history, medical history, and treatment before admission (*p* > 0.05). However, there was a statistically significant difference in the occupational composition between the two groups (*χ*^2^ = 6.012, *p* = 0.049) (Table 5).

**Table 5:** Comparison of basic information of patients with different length of stay [Example (%)].

Hospital stay duration	Clinical data	Total	Category		*χ*^2^/*z*	*p*
			Stable nucleic acid CT values (660)	Fluctuating nucleic acid CT values (267)		
Group A		418	233	185		
	Age		39 [30, 52]	43 [31, 52]	−1.262	0.207
	Sex				0.272	0.602
	Male	254 (60.77)	139 (59.66)	115 (62.16)		
	Female	164 (39.23)	94 (40.34)	70 (37.84)		
	Occupation				0.641	0.726
	Low-risk occupation	40 (9.57)	20 (8.58)	20 (10.81)		
	Medium risk occupation	172 (41.15)	98 (42.06)	74 (40.00)		
	High-risk occupation	206 (49.28)	115 (49.36)	91 (49.19)		
	Vaccine				0.180	0.670
	Unvaccinated	27 (6.46)	14 (6.01)	13 (7.03)		
	Vaccinated	391 (93.54)	219 (93.99)	172 (92.97)		
	Previous history				1.820	0.177
	Yes	70 (16.79)	34 (14.59)	36 (19.57)		
	No	347 (83.21)	199 (85.41)	148 (80.43)		
	Treatment before admission				2.423	0.120
	No	368 (88.04)	200 (85.84)	168 (90.81)		
	Yes	50 (11.96)	33 (14.16)	17 (9.19)		
Group B		509	427	82		
	Age		39 [28, 52]	34 [28, 48]	−1.035	0.301
	Sex					
	Male	296 (58.15)	249 (58.31)	47 (57.32)	0.028	0.867
	Female	213 (41.85)	178 (41.69)	35 (42.68)		
	Occupation				6.012	0.049*
	Low-risk occupation	45 (8.84)	32 (7.49)	13 (15.85)		
	Medium risk occupation	270 (53.05)	229 (53.63)	41 (50.00)		
	High-risk occupation	194 (38.11)	166 (38.88)	28 (34.15)		
	Vaccine					
	Unvaccinated	36 (7.11)	31 (7.31)	5 (6.10)	0.153	0.696
	Vaccinated	470 (92.89)	393 (92.69)	77 (93.90)		
	Previous history				0.020	0.888
	Yes	60 (11.88)	50 (11.79)	10 (12.35)		
	No	445 (88.12)	374 (88.21)	71 (87.65)		
	Treatment before admission				0.621	0.431
	No	459 (90.18)	387 (90.63)	72 (87.80)		
	Yes	50 (9.82)	40 (9.37)	10 (12.20)		

*p < 0.05, the difference is statistically significant.

### 3.5 Clinical data and laboratory examinations

In Group A, there was a statistically significant difference in red blood cell count (*z* = −2.800, *p* = 0.005). However, there were no statistically significant differences between the two groups in terms of TCM symptoms at admission, fever after admission, comprehensive evaluation of medical records, TCM diagnosis at discharge, and various indicators (temperature, breath, heart rate, systolic blood pressure, diastolic blood pressure, oxygen saturation, total bilirubin, direct bilirubin, creatinine, white blood cell count, bacterial count, hemoglobin, platelet count, albumin, glutamic-pyruvic transaminase, glutamic oxaloacetic transaminase, blood urea nitrogen, NLR) (*p* > 0.05). In Group B, there were statistically significant differences in direct bilirubin (*z* = −2.605, *p* = 0.009) and platelet count (*z* = −2.988, *p* = 0.003). However, there were no statistically significant differences between the two groups in terms of TCM symptoms at admission, fever after admission, comprehensive evaluation of medical records, TCM diagnosis at discharge, and various indicators (temperature, breath, heart rate, systolic blood pressure, diastolic blood pressure, oxygen saturation, total bilirubin, red blood cell count, creatinine, white blood cell count, bacterial count, hemoglobin, albumin, glutamic-pyruvic transaminase, glutamic oxaloacetic transaminase, blood urea nitrogen, NLR) (*p* > 0.05) (Tables 6, 7).

**Table 6:** Comparison of clinical data of patients with different length of stay after admission [P25, P75].

Hospital stay duration	Clinical data	Total	Category		*χ*^2^/*z*	*p*
			Stable nucleic acid CT values (660)	Fluctuating nucleic acid CT values (267)		
Group A		418	233	185		
	TCM symptom				0.600	0.438
	No	68 (16.27)	35 (15.02)	33 (17.84)		
	Yes	350 (83.73)	198 (84.98)	152 (82.16)		
	Fever after admission				1.188	0.276
	Yes	111 (62.01)	61 (58.65)	50 (66.67)		
	No	68 (37.99)	43 (41.35)	25 (33.33)		
	Comprehensive evaluation of admission records				2.380	0.123
	Light	350 (98.04)	202 (99.02)	148 (96.73)		
	Ordinary	7 (1.96)	2 (0.98)	5 (3.27)		
	Discharged TCM diagnosis				3.429	0.180
	Lung meridian stagnation fire syndrome	215 (51.44)	113 (48.50)	102 (55.14)		
	Evidence of cold and dampness invading outside	189 (45.22)	114 (48.93)	75 (40.54)		
	Others	14 (3.35)	6 (2.58)	8 (4.32)		
Group B		509	427	82		
	TCM symptom				0.283	0.595
	No	65 (12.77)	56 (13.11)	9 (10.98)		
	Yes	444 (87.23)	371 (86.89)	73 (89.02)		
	Fever after admission				0.561	0.454
	Yes	81 (54.36)	68 (53.13)	13 (61.90)		
	No	68 (45.64)	60 (46.88)	8 (38.10)		
	Comprehensive evaluation of admission records				0.022	0.883
	Light	440 (98.43)	368 (98.40)	72 (98.63)		
	Ordinary	7 (1.57)	6 (1.60)	1 (1.37)		
	Discharged TCM diagnosis				1.045	0.593
	Lung meridian stagnation fire syndrome	305 (59.92)	252 (59.02)	53 (64.63)		
	Evidence of cold and dampness invading outside	186 (36.54)	159 (37.24)	27 (32.93)		
	Others	18 (3.54)	16 (3.75)	2 (2.44)		

*p < 0.05, the difference is statistically significant.

**Table 7:** Comparison of laboratory indicators of patients with different length of stay [P25, P75].

Hospital stay duration	Various indicators	Category		*z*	*p*
		Stable nucleic acid CT values (660)	Fluctuating nucleic acid CT values (267)		
Group A		233	185		
	Temperature	36 [36, 37]	36 [36, 37]	−0.483	0.629
	Breath	18 [18, 20]	18 [18, 20]	−0.456	0.648
	Heart rate	90 [80, 101.5]	88 [80, 100]	−1.812	0.070
	Systolic blood pressure	122 [120, 130]	120 [120, 130]	−1.281	0.200
	Diastolic blood pressure	80 [75, 84]	80 [71, 82]	−0.561	0.575
	Blood oxygen saturation	98 [96, 99]	98 [96, 99]	−0.184	0.854
	Total bilirubin	11 [8, 14]	10 [7, 14]	−0.964	0.335
	Direct bilirubin	3 [2, 4]	3 [2, 4]	−0.892	0.372
	Creatinine	67 [53, 78]	68 [51, 79]	−0.083	0.934
	Red blood cell count	6 [2, 15]	4 [2, 12]	−2.800	0.005*
	White blood cell count	8 [4, 21.5]	7 [4, 16]	−0.900	0.368
	Bacterial count	21 [4, 164]	12 [4, 135.5]	−0.920	0.358
	Hemoglobin	144 [130, 158]	146 [131, 157.5]	−0.420	0.674
	Platelet count	195 [166.5, 237.5]	191 [159.5, 237]	−0.984	0.325
	Albumin	43 [40, 45]	42 [39, 45]	−1.037	0.300
	Glutamic-pyruvic transaminase	20 [11, 34]	22 [12, 38]	−0.928	0.353
	Glutamic oxaloacetic transaminase	21 [16, 28]	22 [16.5, 29.5]	−0.769	0.442
	Urea nitrogen	4 [3, 5]	4 [3, 5]	−0.479	0.632
	NLR (Neutral Particle Count/Lymphocyte Count)	1.38 [0.975, 2.225]	1.35 [0.89, 2.0]	−1.131	0.258
Group B		427	82		
	Temperature	36 [36, 37]	36 [36, 37]	−0.508	0.611
	Breath	18 [18, 20]	18.5 [18, 20]	−0.117	0.907
	Heart rate	90 [80, 100]	86 [78, 98]	−1.778	0.075
	Systolic blood pressure	120 [118, 128]	120 [116, 127.5]	−0.323	0.746
	Diastolic blood pressure	80 [72, 82]	79 [70, 81.25]	−0.789	0.430
	Blood oxygen saturation	98 [97, 99]	98 [97, 99]	−0.589	0.556
	Total bilirubin	10 [0, 14]	10 [8, 14]	−1.417	0.157
	Direct bilirubin	3 [0, 4]	3 [2, 4]	−2.605	0.009*
	Creatinine	58 [0, 73]	61.5 [47.75, 76]	−1.735	0.083
	Red blood cell count	4 [2, 13]	6 [1, 13]	−1.035	0.301
	White blood cell count	7 [4, 22]	7 [4, 17]	−0.575	0.565
	Bacterial count	22 [5, 150]	20 [4, 170.25]	−0.123	0.902
	Hemoglobin	144 [132, 158]	141 [126.5, 157]	−1.442	0.149
	Platelet count	213 [172, 255]	186.5 [157.5, 226.25]	−2.988	0.003*
	Albumin	42 [0, 44]	42 [39, 44.25]	−1.665	0.096
	Glutamic-pyruvic transaminase	17 [0, 32]	18.5 [12, 31.25]	−1.036	0.300
	Glutamic oxaloacetic transaminase	19 [0, 25]	20 [15, 26.25]	−1.291	0.197
	Urea nitrogen	4 [0, 5]	4 [3, 5]	−1.026	0.305
	NLR (Neutral Particle Count/Lymphocyte Count)	1.34 [0.93, 1.82]	1.3262 ± 0.90650	−1.243	0.214

*p < 0.05, the difference is statistically significant.

### 3.6 Logistic regression analysis of factors influencing nucleic acid CT value fluctuations in Group B

Factors that showed statistically significant differences in the univariate analysis of Group B, including occupational composition, direct bilirubin, and platelet count, were included in a binary logistic regression model. Nucleic acid CT value fluctuation was the dependent variable, and the three aforementioned variables, along with age as a forced variable, were entered into the model using a forward: LR method. The results showed that occupational composition (high-risk occupation, *p* = 0.005, OR = 3.159) and direct bilirubin (*p* = 0.029, OR = 0.997) were significantly associated with nucleic acid CT value fluctuations. None of the other indicators were independent risk factors for nucleic acid CT value fluctuations (*p* > 0.05) (Table 8).

**Table 8:** Logistic regression analysis of fluctuations in nucleic acid CT values of patients in group B.

Risk factors	OR (95%CI)	*p*
Age	0.344 (0.971–1.003)	0.050
Occupation		
Low-risk occupation	–	0.015
Medium risk occupation	1.103 (0.645–1.889)	0.720
High-risk occupation	3.159 (1.418–7.035)	0.005*
Direct bilirubin	0.997 (0.994–1.000)	0.029*

*p < 0.05, the difference is statistically significant.

## 4 Discussion

Since the onset of the COVID-19 pandemic in 2019, more than 5 years have elapsed. During this period, the virus has evolved numerous variants, posing a significant public health challenge globally. Patients who test negative for nucleic acid may still be affected by COVID-19 (40). Among the variant strains, the Omicron variant has shown a decrease in pathogenicity but possesses strong infectivity and rapid transmission, causing significant impact in certain regions (41, 42). The fluctuation in nucleic acid CT values has undoubtedly posed challenges to the normalization of epidemic prevention and control, severely affecting people’s normal lives and mental well-being (43). Studies have found that approximately one-fourth of patients experience fluctuations in nucleic acid CT values (44), which may persist or recur over a prolonged period (45). Significant correlations have been observed between nucleic acid CT values and factors such as length of hospital stay, vaccination status (46), age (47), and albumin levels (48).

In this study, there was a statistically significant difference in the occupational composition between the group with nucleic acid CT value fluctuations and the group without fluctuations (*χ*^2^ = 7.043, *p* = 0.03). It was found that the viral shedding time of healthcare workers was prolonged (49), and the probability of nucleic acid CT value fluctuations increased with the nature of their occupation (39). The likelihood of contracting COVID-19 is inevitably associated with the workplace and occupation (50, 51). Univariate analysis of nucleic acid CT value fluctuations revealed a significant correlation with hospitalization duration (*z* = −12.672, *p* = 0.000), consistent with previous research that showed a correlation between nucleic acid CT value fluctuations and hospitalization duration (*p* = 0.00018, 13). Therefore, nucleic acid CT fluctuations are an important factor related to length of stay (38, 52). This suggests a significant correlation between nucleic acid CT value fluctuations and hospitalization duration, and binary logistic regression analysis results indicate a positive correlation between nucleic acid CT value fluctuations and hospital stay duration, indicating that the longer the hospitalization duration, the greater the risk of nucleic acid CT value fluctuations. Some studies have indicated that older adult patients, due to their weaker immune system and higher prevalence of underlying diseases such as hypertension and diabetes, have a lower clearance rate and tolerance to the virus, making them more prone to nucleic acid CT value fluctuations (53, 54). However, the results of this study showed no significant correlation between nucleic acid CT value fluctuations and age or medical history, which is inconsistent with other related studies (39, 48, 55). Given the weakened immune response and decreased organ reserve capacity in the older adult, leukocyte count and C-reactive protein levels are associated with nucleic acid CT values (56). Therefore, it is crucial to pay attention to this population to prevent the occurrence of nucleic acid CT value fluctuations, which can cause irreversible damage to the body, aligning with the research on nucleic acid CT value fluctuations in the Northeast region (57). This suggests that the influence of gender composition on nucleic acid CT value fluctuations may be region-specific, while age may be related to the fact that the subjects in this study were predominantly young and middle-aged individuals.

Although this study found no statistically significant difference between the fluctuation of patient nucleic acid CT values and vaccination history, COVID-19 vaccines have shown high efficacy and good tolerability against the original strain and related variants. Both two-dose and three-dose regimens provide good protection against Omicron, although efficacy may decrease over time and compared to other strains (58–60). This research does not establish a significant correlation between the fluctuations in the nucleic acid CT values and vaccination. However, it still emphasizes the effectiveness of COVID-19 vaccination in preventing and controlling the Omicron variant, and encourages people to get vaccinated to prevent reinfection. Nucleic acid CT values have certain relationships with factors such as NLR, white blood cell count, C-reactive protein, and platelet count (56, 61–63). This study shows significant correlations between nucleic acid CT fluctuation and patient creatinine, platelet count, alanine aminotransferase, and aspartate aminotransferase, but no significant correlation with white blood cell count, NLR (neutrophil count/lymphocyte count), or hemoglobin. This suggests that the strength of a patient’s immune system and the normal function of the liver are related to nucleic acid CT fluctuation. Binary logistic regression analysis reveals that platelet count (*p* = 0.019, OR = 0.996) is an independent factor influencing nucleic acid CT value fluctuation and is negatively correlated, which is consistent with previous studies. This indicates that the stronger the patient’s immune system, the lower the risk of the fluctuation of nucleic acid CT values fluctuation. Studies have found a high risk of arrhythmias in COVID-19 patients (64). The reasons for arrhythmias in COVID-19 are still unclear and may be a direct effect of the virus or adverse reactions to medications used for treatment. This is consistent with the statistically significant difference in nucleic acid CT fluctuation and heart rate (*z* = −2.224, *p* = 0.026) observed in this study. Binary logistic regression analysis shows that heart rate (*p* = 0.036, OR = 0.979) is an independent factor influencing nucleic acid CT value fluctuation and is negatively correlated. This suggests that paying attention to cardiovascular health and receiving anti-arrhythmia treatment has a significant effect on preventing nucleic acid CT fluctuation. Further research has found that regular aerobic exercise and its effect on heart rate are consistent with the median heart rate results in both groups (65). This indicates that regular aerobic exercise, which increases myocardial oxygen consumption and enhances immune function, is an effective approach to control nucleic acid CT fluctuation.

The study found that red blood cell count is an influencing factor for nucleic acid CT fluctuation in Group A, which is consistent with the well-established manifestation of anemia in COVID-19 infection (66–68). Although anemia does not directly affect the mortality rate of COVID-19 patients, its impact on the older adult should not be overlooked. Due to their diminished immunity and general frailty, older adult individuals are less likely to recover quickly from COVID-19, leading to prolonged hospital stays and severely diminishing their quality of life. In Group B, occupational composition, direct bilirubin, and platelet count are influencing factors for nucleic acid CT fluctuation, and binary logistic regression analysis shows that occupational composition and direct bilirubin are independent factors influencing nucleic acid CT value fluctuation and are positively and negatively correlated, respectively. This indicates that short-term hospitalized patients in high-risk occupations are more prone to nucleic acid CT fluctuation, which is consistent with the factors influencing nucleic acid CT fluctuation mentioned earlier and further emphasizes the higher risk of nucleic acid CT fluctuation in short-term hospitalized patients engaged in high-risk occupations. For short-term hospitalized patients, the lower the direct bilirubin within the normal range, the higher the risk of nucleic acid CT fluctuation, which is consistent with research showing that direct bilirubin prolongs viral shedding time in Omicron patients (69).

## 5 Conclusion

Based on the retrospective study, it was found that the occupation composition, hospital stay duration, heart rate, creatinine, platelet count, alanine transaminase, and aspartate transaminase were factors influencing the fluctuation of nucleic acid CT values in COVID-19 patients. Among them, hospital stay duration, platelet count, and heart rate were identified as independent factors affecting the fluctuation of nucleic acid CT values, with a positive correlation observed with the hospital stay duration and a negative correlation with platelet count and heart rate. Analyzing the relationship between hospital stay duration and the fluctuation of nucleic acid CT values, it was determined that red blood cell count was a factor influencing the fluctuation of nucleic acid CT values fluctuation in Group A patients, while occupation composition, direct bilirubin, and platelet count were factors influencing the fluctuation of nucleic acid CT values fluctuation in Group B patients. In Group B, occupation composition and direct bilirubin were identified as independent factors affecting the fluctuation of nucleic acid CT values, positively correlated with occupational risk and negatively correlated with direct bilirubin.

Therefore, enhancing patients’ immune function, increasing physical exercise to improve myocardial oxygen consumption, reducing hospital stay duration, and closely monitoring liver function to prevent liver function abnormalities at the initial stage of hospitalization are effective measures for controlling the fluctuation of nucleic acid CT values in COVID-19 patients. However, due to the limited clinical data collected, these findings may not comprehensively and accurately reflect the factors influencing the fluctuation of nucleic acid CT values in COVID-19. Additionally, the regional limitations of the data collection may not represent all regions. Therefore, future research should adopt large sample sizes and a multicenter approach to provide reliable evidence for comprehensive and accurate prediction of nucleic acid CT value fluctuation in COVID-19, thus supporting data-driven strategies for epidemic prevention and control in the post-pandemic era.

## Data availability statement

The raw data supporting the conclusions of this article will be made available by the authors, without undue reservation.

## Ethics statement

The studies involving humans were approved by the Ethics Committee of the Affiliated Hospital of Changchun University of Traditional Chinese Medicine (Approval No: CCZYFYLL2022 Approval Letter-020; CCZYFYLL2022 Approval Letter-021). The studies were conducted in accordance with the local legislation and institutional requirements. Written informed consent for participation was not required from the participants or the participants’ legal guardians/next of kin in accordance with the national legislation and institutional requirements.

## Author contributions

ZC: Writing – original draft. FS: Data curation, Writing – original draft. HD: Data curation, Writing – original draft. ZT: Data curation, Writing – original draft. YC: Conceptualization, Data curation, Writing – review & editing. WY: Data curation, Writing – review & editing. SH: Writing – review & editing, Conceptualization, Funding acquisition. LS: Conceptualization, Writing – review & editing, Funding acquisition.
